# Are Household Potato Frying Habits Suitable for Preventing Acrylamide Exposure?

**DOI:** 10.3390/foods9060799

**Published:** 2020-06-17

**Authors:** Marta Mesias, Cristina Delgado-Andrade, Francisco J. Morales

**Affiliations:** Institute of Food Science, Technology and Nutrition, ICTAN-CSIC, José Antonio Novais 10, 28040 Madrid, Spain; cdelgado@ictan.csic.es (C.D.-A.); fjmorales@ictan.csic.es (F.J.M.)

**Keywords:** consumers, domestic habits, french fries, frying habits, households, oil, acrylamide

## Abstract

A survey was conducted of 730 Spanish households to identify culinary practices which might influence acrylamide formation during the domestic preparation of french fries and their compliance with the acrylamide mitigation strategies described in the 2017/2158 Regulation. Spanish household practices conformed with the majority of recommendations for the selection, storing and handling of potatoes, with the exception of soaking potato strips. Olive oil was the preferred frying oil (78.7%) and frying pans were the most common kitchen utensils used for frying (79.0%), leading to a higher oil replacement rate than with a deep-fryer. Although frying temperature was usually controlled (81.0%), participants were unaware of the maximum temperature recommended for preventing acrylamide formation. For french fries, color was the main criteria when deciding the end-point of frying (85.3%). Although a golden color was preferred by respondents (87.3%), color guidelines are recommended in order to unify the definition of “golden.” The results conclude that habits of the Spanish population are in line with recommendations to mitigate acrylamide during french fry preparation. Furthermore, these habits do not include practices that risk increasing acrylamide formation. Nevertheless, educational initiatives tailored towards consumers would reduce the formation of this contaminant and, consequently, exposure to it in a domestic setting.

## 1. Introduction

Chemical process contaminants are substances formed when foods undergo chemical changes during processing, including heat treatment, fermentation, smoking, drying and refining [1]. Although necessary for making food edible and digestible, heat treatment can have undesired consequences leading to the formation of heat-induced contaminants such as acrylamide [2]. Acrylamide is a chemical process contaminant formed when foods containing free asparagine and reducing sugars are cooked at temperatures above 120 °C in low moisture conditions [3]. It is mainly formed in baked or fried carbohydrate-rich foods as the relevant raw materials contain its precursors. These include cereals, potatoes and coffee beans. In 1994, acrylamide was classified by the International Agency for Research on Cancer as being probably carcinogenic to humans (group 2A) [4]. In 2015, the European Food Safety Authority (EFSA) confirmed that the presence of acrylamide in foods is a public health concern, requiring continued efforts to reduce its exposure [3].

Fried potato products are the main contributor to total dietary acrylamide exposure, especially amongst young people [3]. The recent 2017/2158 Regulation [5] on benchmark levels for reducing the presence of acrylamide in foods and the acrylamide toolbox compiled by Food Drink Europe [6] include specific mitigation strategies for decreasing the presence of acrylamide in foodstuffs. The category of fried potato products is divided between two subcategories. The first category describes potato-based snacks, whilst the second describes french fries and other cut potato products. When fried potato products are made from fresh potatoes, mitigation measures are focused on the following factors: selection of suitable potato varieties, acceptance criteria based on quality, potato storage and transport, recipe and process design. In addition, information for end users can be supplied in packaging if potatoes are intended to be fried at home. These recommendations are aimed at controlling the precursor levels in fresh tubers (mainly reducing sugar content), frying temperature and the color of the final product. Further, they are fundamentally focused on the industrial sector [5,6]. As a result of following these measures, acrylamide levels in potato chips have decreased in recent years, demonstrating that mitigation strategies are being successfully applied to industrial potato crisps [7,8,9]. However, these strategies cannot be directly extended to private domestic settings as the main variables accounting for the cooking process vary between households and even between individuals [10]. Consumers may significantly influence their dietary acrylamide exposure through their purchase choices and the selection of culinary methods for cooking food, amongst other factors [11,12]. The EFSA has reported that home-cooking behaviors for potato frying lead to variations of up to 80% total dietary acrylamide exposure.

Several initiatives have been driven by different national food safety authorities with the aim of helping consumers understand how they can minimize acrylamide exposure when cooking at home. Examples of this include the “go for gold” campaign by the Food Standards Agency (FSA) [11] and the “golden but not toasted” campaign by the Spanish Food Safety Agency [13]. Both campaigns intended to educate audiences on how to identify the golden color that is characteristic of a healthier fry and lower acrylamide content in fried potatoes. Despite these campaigns, the population may not be applying mitigation measures during the preparation of french fries at home. They may even be unaware that acrylamide is an issue and that its formation can be reduced during frying. In June 2019, the EFSA published the last special Eurobarometer on risk perceptions, which included the results of a survey of 27,655 respondents from different social and demographic groups around Europe [14]. The survey showed that when considering commonly known food safety related topics, European citizens were most concerned about antibiotic residues; hormones or steroids in meat (44%); pesticide residues in food (39%); environmental contaminants in fish, meat or dairy products (37%) and additives such as colorants and preservatives or flavors used in food or beverages (36%). Process contaminants in foods, including acrylamide, were not listed amongst these topics.

Within this framework, the aim of the present work was to explore domestic practices for the preparation of french fries in Spanish households using a survey of domestic culinary habits and consumer preferences. The degree of compliance with mitigation strategies for reducing acrylamide formation in french fries was then evaluated, whilst also identifying unwanted practices that could be modified through re-education to favor healthier frying habits amongst the Spanish population. To our knowledge, this is the first survey conducted in a Spanish population with a focus on french fry preparation habits.

## 2. Materials and Methods

### 2.1. Study Design

The experimental design was based on similar studies in the literature [15,16,17]. The questionnaire was supervised by researchers from the Food Innovation research team at the Polytechnic University of Valencia (Valencia, Spain). Firstly, the survey was piloted with 20 individuals via one-on-one interviews. This was done to identify specific focal points for questionnaire development and to make the necessary adjustments based on respondent feedback. Following this, the questionnaire was drafted and validated with 35 subjects. The survey included thirty-two questions divided into seven different topics. These were:

- Socio-demographic characteristics: gender, age group, nationality, type of household, number of individuals at home and number of individuals under 18 years old at home.
- Culinary habits: cooking experience.
- French fry consumption: characteristics of potatoes intended for frying (fresh or frozen, par-fried), frequency of french fry consumption and the way in which they are consumed.
- Fresh potato characteristics: place of purchase and type of fresh tubers (in-season or stored, washed or unwashed, bulk or bagged, labelled as “special for frying,” etc.), geographical origin, botanical variety and place of storage at home.
- Practices at the pre-frying stage: peeling, washing, soaking, adding salt and cutting preferences.
- Practices at the frying stage: kitchen utensil, frying oil characteristics, frying temperature, subjective ratio between the amount of food with respect to the dimensions of the frying utensil, defrosting prior to par-frying potatoes or frying from frozen and frying cycles.
- Post-frying practices: criterion for establishing the frying end-point, color and texture preferences; method for removing oil from fried food; criteria for stopping oil use in other frying cycles; cleaning procedures for oil reuse and oil storage.

Questions were structured according to check boxes with unique or multiple possible responses. The questionnaire included a brief introduction about the aim of the study and participants gave their consent to use their data in the research (Supplementary Material: Survey on household habits in relation to frying potatoes).

### 2.2. Data Collection

The present study was conducted with 730 respondents, using both online (*n* = 421) and paper-based (*n* = 309) questionnaires. A web-based survey was launched via an internet platform through Google form links (Melbourne, Australia), and paper-based questionnaires were directly distributed to respondents. Questionnaire data were included when more than 75% of the survey was completed and no more than two questions per topic were missing. Both datasets were merged for analysis since the approach through which information was compiled was not a limiting source of variability for analysis. The selection of participants was limited to adults who consume or prepare french fries at home. The main channels for questionnaire distribution were schools, consumer associations, universities, food associations, research centers, culinary centers, etc. Participants agreed to the anonymous use of their data for the study.

### 2.3. Data Processing

Outputs from the online and paper-based questionnaires were merged and compiled in an excel spreadsheet format. Data processing was performed using SPSS version 23.0 (SPSS Inc., Chicago, IL, USA). Categorical data were expressed as frequencies and percentages.

## 3. Results and Discussion

### 3.1. Characteristics of the Participants

**Figure 1:**
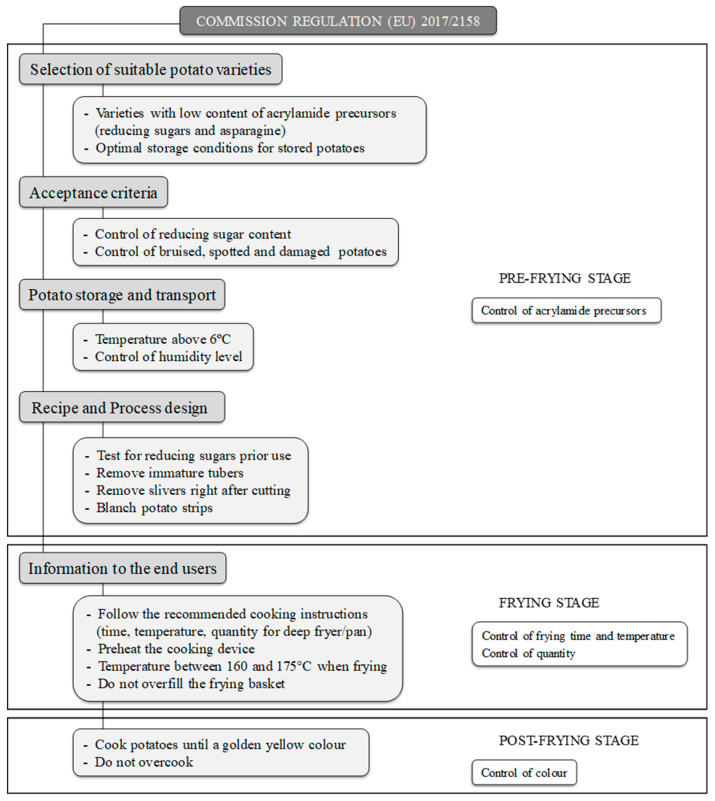
Mitigation measures referred to french fries made from raw potatoes according to the 2017/2158 Commission Regulation (EU) adapted for domestic habits.

The present study aimed to evaluate the adhesion of potato frying habits in Spanish households to recommendations for mitigating acrylamide formation provided by the European 2017/2158 Regulation [5]. It has to be pointed out that the purpose was not to give advice on acrylamide and frying practices or to assess the population’s knowledge of these issues but to evaluate the domestic habits for the preparation of french fries and to compare them with the recommendations. Figure 1 depicts the critical action points for reducing acrylamide formation during the preparation of fried potatoes from fresh tubers according to three stages (pre-frying, frying, post-frying). An ad-hoc survey of culinary practices for the preparation of fried potatoes in households was conducted with Spanish adults. To obtain a representative and diverse number of respondents and minimize response bias, the survey included both online and paper-based questionnaires [18]. The web-based format allows for a broader dissemination, being less time demanding and less expensive, whilst paper-based questionnaires can be distributed amongst people who are not familiar with the internet or have limited access to digital platforms.

For the cross-sectional population survey, a cohort of seven hundred and forty-eight volunteers was recruited from 47 of the 50 provinces of Spain. Eighteen participants did not complete the paper-based questionnaire and their data were withdrawn. Thus, the final number of responders included in the study was 730. Four hundred and twenty-one individuals filled out the online questionnaire and 309 completed the paper version. Participants were adults who usually prepare or consume french fries at home. The sociodemographic characteristics of the participants are summarized in Table 1. Despite that currently, 11% of the population living in Spain is foreign [19], 97.8% of the surveyed were of Spanish nationality. Participants were mostly female (73.2%), and aged between 36 and 55 years old (54.1%). With regards to the level of culinary experience, 72.3% of women considered themselves to have high expertise in contrast to 52.1% of men. This distribution corroborates gender differences reported in the ENHALI-2012 study [20], identifying a higher participation of women in the domestic activity of food preparation. Households were constituted by two (29.7%), three (26.0%) or four or more individuals (34.3%), with most not being comprised of any individual aged under 18 years (59.5%). The majority of participants live with a partner (70.7%), with 45.1% living with children and relatives, and 25.6% being couples without children. The household profile is consistent with the typology described for the Spanish population, with smaller and one-person households made up of a young person or an independent adult predominating [21].

### 3.2. French Fry Consumption

Most respondents (85.3%) usually buy fresh tubers to prepare french fries at home. In total, 21 (2.9%) reported preparing only frozen par-fried potatoes, with 84 (11.5%) using both foods indistinctly (Table 2). This finding agrees with the data described in the food consumption report in Spain 2018. This indicated that fresh tubers and frozen par-fried potatoes represent 72.7% and 3.4%, respectively, of all potatoes bought by the Spanish population [21]. A total of 43.5% of respondents affirmed consuming french fries several times a month (monthly), whilst 35.9% reported a consumption of several times a week (weekly). A lower percentage eats these products only on exceptional occasions (19.7%) and only two individuals indicated a daily consumption (0.3%). With regards to consumption preferences, french fries make up the side dish for other foods (97.1%) such as meat (79.7%); fish (23.8%); vegetables (8.9%) and other foods including fried eggs, sausages, croquettes, etc. (47.6%).

**Figure 2:**
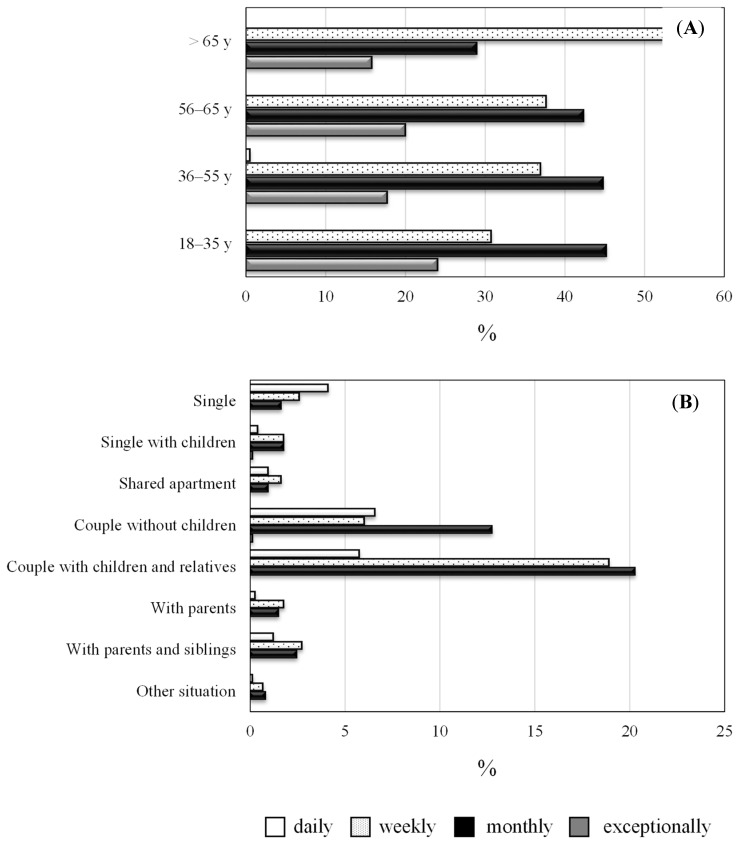
Distribution of french fry consumption according to participants’ age (expressed in relative amounts within each age group) (**A**) and the type of household (expressed in absolute amounts for all respondents (**B**): 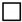 daily, 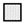 weekly, 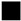 monthly and 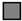 exceptionally).

Potato chips or french fries are a cause of concern due to their high fat and energy content. This is associated with a higher incidence of diseases such as obesity, high blood pressure and/or hypercholesterolemia [22]. For this reason, although frying is an ancestral and popular culinary technique and a typical way of cooking in the Mediterranean diet [23], nutritional recommendations limit this culinary practice to prevent its associated health complications. The relationship was evaluated between french fry consumption and both age of and household type. The most significant difference was observed in people older than 65 years of age who usually consume potatoes several times a week (weekly), with the frequency of consumption in other age groups predominantly being several times a month (monthly) (Figure 2A). Households with a higher potato consumption were typically formed by couples with children, followed by couples without children. These groups predominantly reported a monthly or weekly consumption (Figure 2B). Similarly, in Spain, a higher potato consumption corresponds to households made up of middle-aged and older children, single-parent households and households made up of adult couples without children [21].

### 3.3. Fresh Potato Characteristics

The survey showed that fresh tubers are mainly bought in supermarkets (48.1%) and neighborhood grocery stores (44.1%), followed by hypermarkets (29.3%) and markets (16.2%) (Table 2). This agrees with purchase habits described for the global Spanish population [21]. Respondents are generally interested in knowing the geographical origin and/or botanical variety of the potato, although 38.1% are not interested in this aspect. Some of the common varieties used in Spain to prepare french fries are Monalisa, Caesar, Milva and Agria [12]. Potatoes are normally bought in bulk rather than bagged (36.1 vs. 28.1), and are washed (43.3%) and fresh harvested (in-season) (49.1%). However, some respondents expressed not being concerned about the type of potato purchased in relation to its presentation (6.0%) and seasonality (24.1%).

**Figure 3:**
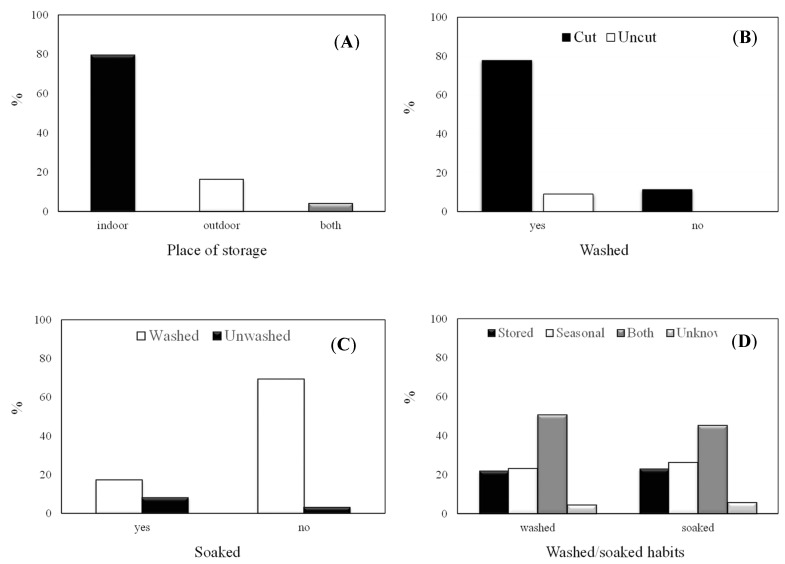
Handling habits of fresh tubers: place of storage (**A**), washing-related habits (cut or uncut potatoes) (**B**), soaking-related habits (washed or unwashed potatoes) (**C**), washing and soaking-related habits (stored and seasonal potatoes) (**D**).

Five hundred and seventeen individuals (70.8%) indicated that they do not buy “special frying potatoes,” with a further 98 (13.4%) not being aware of this type of classification. According to commercial quality norms for potatoes for consumption in the Spanish market, commercial potatoes must be identified according to product type (variety, uncalibrated/calibrated, in-season/stored), origin (country and, optionally, regional or local production area or national denomination) and commercial characteristics (category, net weight, batch, caliber/size, recommended culinary use, etc.). For bagged potatoes, the name, trade name or designation of the packer and/or shipper or seller should also be identified [24]. Regarding the place of storage, 79.6% store potatoes indoors, whilst 4.0% keep their potatoes both indoors and outdoors (Figure 3A) The UK Food Standards Agency (FSA) drew up a report which aimed to provide information on actual domestic cooking and french fry preparation practices in the UK. The report indicated that the majority of individuals in the UK usually store their potatoes outside of the fridge, such as in a kitchen cupboard, garage or utility room, prior to preparing and cooking them [11]. This is similar to the Spanish habits observed in the present study.

For the preparation of fried potato products based on raw potatoes, the 2017/2158 Regulation recommends the selection of suitable potato varieties. This urges a reducing sugar (fructose and glucose) and asparagine content that is as low as possible for regional conditions [5] (Figure 1). Asparagine and reducing sugars are well-known acrylamide precursors, with reducing sugars being the limiting factor of acrylamide-forming potential in potato products [25,26]. Controlling reducing sugar levels is, therefore, currently the primary measure employed by the industry to reduce acrylamide concentration in french fries. This is achieved by selecting potato varieties with a low content of reducing sugars, ensuring that tubers are mature at the time of harvesting, controlling storage conditions and managing humidity to minimize senescent sweetening [6]. Due to the seasonality of this commodity, reducing sugar content varies depending on whether the potato is fresh harvested or stored [27]. It is well documented that temperatures below 6 °C for long-term storage, without an adequate re-conditioning step before frying, lead to senescent sweetening as a result of starch hydrolysis [28]. The higher reducing sugar content of stored potatoes promotes the formation of higher levels of acrylamide during the frying process [6]. However, all of these practices must be controlled at both farming and industrial stages in order to provide consumers with optimum potato tubers for helping to mitigate acrylamide formation in the domestic environment. The most important consumer’s decision when purchasing potatoes for the preparation of french fries is to select those that are fresh harvested, whenever possible, and store them indoors, since temperatures inside the home will not promote the mentioned sweetening. On the other hand, although potatoes labelled as “special for frying” could be expected to be the most suitable potatoes for frying, previous research has demonstrated that this commercial label is not always adequate when guiding consumers in the preparation of french fries with low acrylamide content [12].

**Figure 4:**
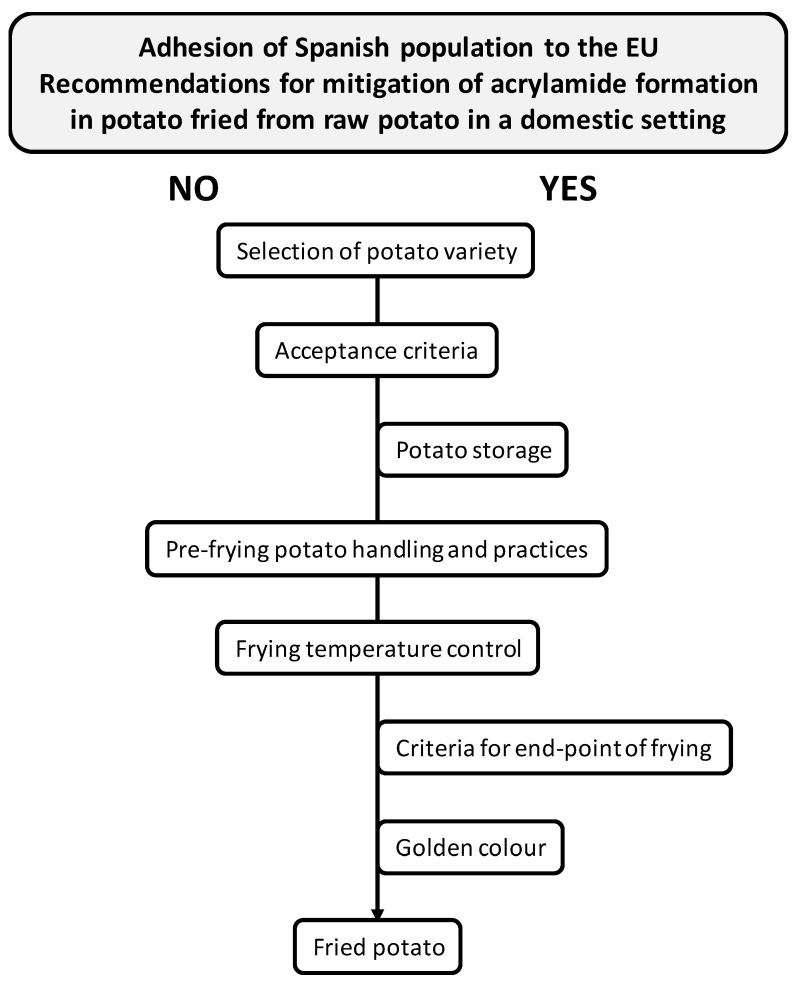
Adherence of the Spanish population to EU recommendations for the mitigation of acrylamide formation in potatoes fried from raw potatoes in a domestic setting [5].

It might be concluded that the Spanish population tends to adhere to acrylamide mitigation strategies relating to storage conditions (Figure 4). However, more information should be provided for consumers to improve their selection of raw materials. This is especially the case for those who do not take the type of potato purchased on the market into consideration, as the acquisition of stored potatoes probably promotes higher acrylamide formation and, thus, a higher reducing sugar content. In addition, use of the “special for frying” label should be supervised by the relevant food authority in order to ensure products exhibit low levels of precursors, in addition to the suitable raw materials intended for frying.

### 3.4. Practices Related to the Pre-Frying Stage

Before frying, most participants peel the potatoes (97.5%) (Table 3) and wash them after peeling (86.8%). Only 43 respondents (5.9%) usually wash tubers before peeling. Amongst the volunteers who wash potatoes (*n* = 634), 66 cut them after washing, whilst the remaining participants do so beforehand (Figure 3B). Most volunteers (*n* = 531) do not soak the potatoes; however, 508 do wash the potato tubers after peeling. In contrast, 126 individuals both wash and soak the potatoes (Figure 3C), with less than 15 min being the most commonly applied soaking time (126 respondents) (Table 3). Both washing and soaking habits are indistinctly applied with stored and in-season tubers (Figure 3D). In order to decrease the sugar content of fresh tubers, especially in the case of stored potatoes, practices such as washing, soaking and/or blanching should be applied [29,30]. The European Commission recommends washing and soaking for 30 min to 2 h in cold water, soaking for a few minutes in warm water or blanching [5] (Figure 1). Although respondents mostly wash potatoes before frying, soaking practices should be included as common habits during french fry preparation in Spain. These practices should be especially promoted when using stored potatoes, as they lead to improved compliance with pre-frying potato handling and practice recommendations (Figure 4). It should be noted that consumers may wash potatoes for reasons other than to mitigate acrylamide formation. Reasons may include heritage habits, hygiene purposes or simply to prevent browning whilst other elements of the meal are prepared. This observation was also made in the FSA report [11].

Other aspects to mention in relation to the pre-frying stage include the addition of salt and the type of potato cut. Salt is mainly added after frying (47.5%) and potatoes are mainly cut into strips (87.9%), followed by slices (21.6%) and cubes (19.5%). In response to this question, participants could provide multiple answers. For this reason, the sum of partial percentages is higher than 100%. Although neither the relevant regulation nor the acrylamide toolbox mention any indication about adding salt before frying, the addition of calcium salts is recommended for the preparation of dough-based potatoes in order to reduce the pH level [5,6]. The presence of NaCl in the food matrix has been described as decreasing the acrylamide content following heat treatment [31]. Thus, as long as the amount of added salt is not high and adheres to health recommendations for salt consumption [32], the population should be encouraged to add salt before frying. With regards to cutting preferences, given that acrylamide is formed on the surface of fried food, controlling thickness is an important factor for preventing its formation. For french fries, it is better to cut in strips than in slices and, in addition, creating thicker strips of potato may reduce acrylamide in french fries [6]. In this sense, several authors have observed an inverse trend between acrylamide levels and french fry thickness [33,34]. The FSA study [11] highlighted, in an English sample, that homemade potato items tended not to be finely chopped, avoiding the risk of additional acrylamide exposure through greater surface area to volume ratios. Similarly, Spanish habits with respect to the type of cut selected for french fries appear to be appropriate, although thickness should be more thoroughly examined.

### 3.5. Practices Related to the Frying Stage

**Figure 5:**
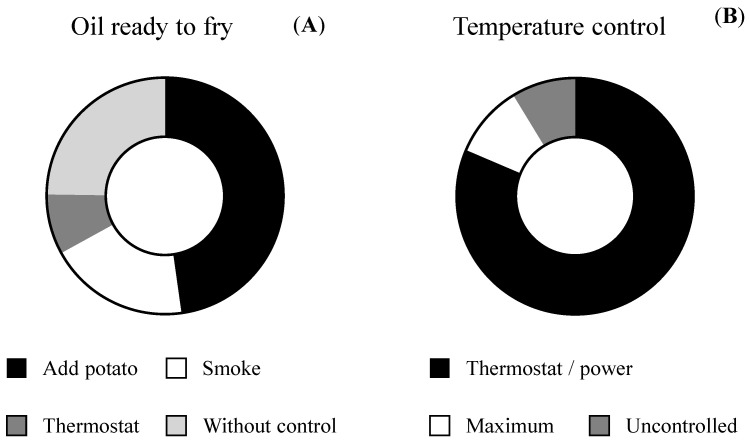
Selection of frying starting point (**A**) and temperature control during french fry preparation (**B**).

Practically all respondents (95.3%) carry out a single frying cycle (Table 3). More than three quarters generally use a frying pan to fry potatoes (79.0%), with 9.2% remarking that the electric fryer was the preferred utensil and 8.6% indicating that they used both types of cookware indistinctly (Table 3). These results contrast those reported by Romero et al. [15], who reported that Spanish University students mainly used electric fryers to prepare french fries (71.3%). Olive oil is the most widely used oil for frying potatoes (indicated by 78.7% of volunteers), with a much smaller percentage selecting sunflower oil as the preferred oil (17.5%). This reflects other profiles reported in previously conducted studies in Spain [35,36]. Respondents’ oil choices are conditioned by their related health properties (468 subjects), taste (375 subjects), oil stability (132 subjects), price (116 subjects) and, to a lesser extent, the appliance used for frying (20 subjects). The majority of participants (73.3%) do not consume oil labelled as “special for frying,” with 144 even being unaware of the existence of these oils. The use of olive oil for frying is associated with healthy habits as it improves the saturated/monounsaturated/polyunsaturated fatty acid profile of food and enriches its fat-soluble vitamin and antioxidant compound content [37]. However, with regards to acrylamide formation, no significant differences have been observed following the preparation of french fries in different edible oils [33]. Recommendations of the acrylamide toolbox and relevant regulation do not make any type of reference to the type of oil to be used for frying, though they do specify the maximum temperatures advised for the oil (Figure 1). Temperature is one of the main factors to determine acrylamide formation in processed foods and, consequently, its control is absolutely essential [33]. Frying temperatures are recommended to be below 175 °C, and as low as possible at all times whilst considering food safety requirements. In addition, it is recommended to preheat the cooking utensil in order to reduce frying time [5,6]. All volunteers preheat the oil and indicated different criteria for checking that oil temperature is ready for frying: adding a potato and observing its behavior (47.5%), smoke emission (smoke point) (18.9%) and controlling the thermostat of the electric fryer (7.9%). At the same time, 24.5% reported not having precise control, adding the food to the frying pan after the oil has warmed up for a period of time (without control) (Figure 5A). The majority of Spanish participants (80.8%) stated that they control the frying temperature by adjusting the thermostat (in the case of using an electric fryer) or the power of the gas flame, glass ceramic or induction plate (in the case of a frying pan) (Figure 5B). From the remaining sample, 68 people always select the maximum temperature, whilst 59 do not control this aspect. Although the majority of individuals reported exercising control of the frying temperature, a temperature below 175 ºC cannot be ensured (Figure 4). Thus, recommendations on the optimal frying temperature for potatoes in order to prevent acrylamide formation should be addressed with the population. Frying temperature may be substantially lower owing to the strong cooling that results from heating the potatoes and water evaporation, further depending on the amount of potato added to the volume of frying oil [38]. Information about the food/frying oil ratio was not obtained in the present survey; however, respondents made inferences based on the surface area of the appliance used. In this sense, the amount of food intended for frying typically amounted to more than half of the dimension of the frying surface (frying pan or frying basket) (33.8%) (Table 3). Thus, not only should recommendations be made for temperature control, but potato/oil ratio should be also considered.

Only 14.5% of participants (*n* = 106) reported consuming frozen par-fried potatoes. Of these, 100 subjects do not defrost the product before frying, following the indications provided on the label of these products. This result could be considered a good practice as the relevant regulation suggests that cooking instructions should be followed for frozen potato products [5].

### 3.6. Practices Related to the Post-Frying Stage

**Figure 6:**
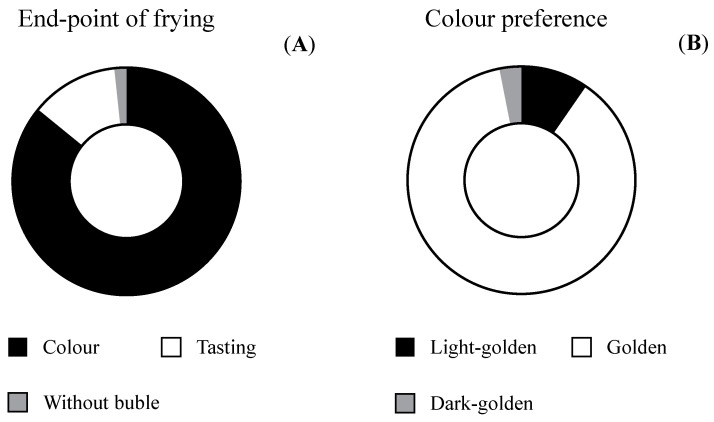
Criteria to decide the frying end-point (**A**) and color preferences in relation to french fries (**B**).

The literature describes a correlation between color and acrylamide formation in french fries [12,39,40,41]. Thus, the decision of food handlers regarding the final color will influence acrylamide exposure from french fries. It is recommendable to cook until a golden (yellow) color is achieved and to avoid overcooking [5,6] (Figure 1). Several educational initiatives have been undertaken to orientate consumers so that they associate the extent of browning in french fries with mitigation strategies for acrylamide at home. Examples include the “go for gold” campaign launched by the UK Food Standards Agency [11] and the slogan “golden but not toasted” by the Spanish Food Safety Agency [13]. Participants in the present study showed an appropriate compliance with recommendations when selecting the color of french fries as the main criteria for choosing the frying end-point (85.3%), with only a minority of respondents stopping the procedure by tasting a sample (11.9%) or when the fried potato stops bubbling in the oil (1.2%) (Figure 6A). Despite golden being the preferred color (87.3%) (Figure 6B), the distribution of color guides providing guidance on the optimal combination of color and low acrylamide levels should not be discarded since previous research by our team has identified the necessity of a clear definition of “golden” amongst consumers [41]. In agreement with these observations, visual checking of color and appearance was the dominant way to decide whether a product was “ready” as desired within a sample of English consumers [11].

**Figure 7:**
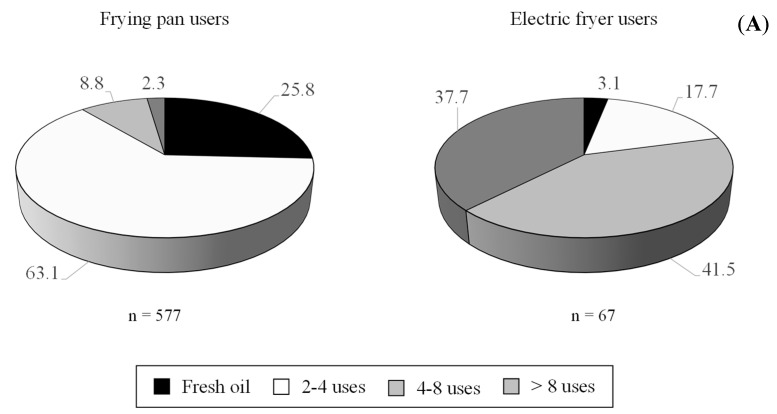

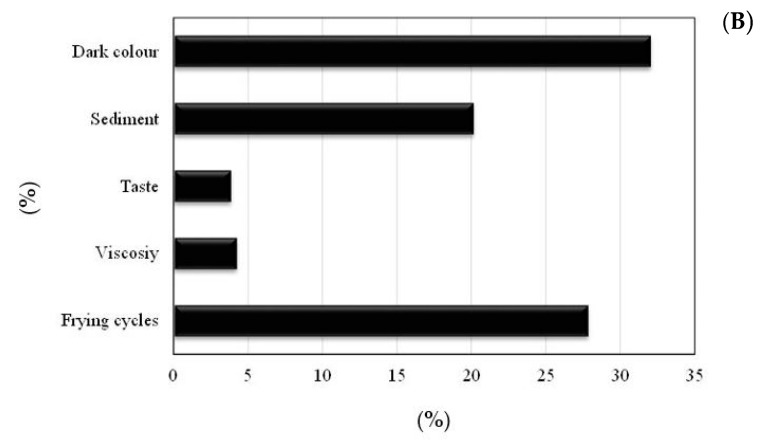
Frequency of use of frying oil (**A**) and reason for changing (**B**).

Another aspect collected in the survey refers to the texture of french fries, with crunchy on the outside and soft on the inside being the preferred characteristic (82.6%) (Table 3). Further, approaches to removing oil from the fried product were considered, with this mainly performed through the application of absorbent paper (58.1%) or paper and rack (21.1%). With regards to the use of frying oil, it is known that oils deteriorate during frying. This leads to changes in the fatty acid composition and retention of other oil-degradation products in fried foods [42]. This fact becomes even more relevant with repeated use. It is, therefore, important to control the number of times that frying oil is reused. In the present survey, amongst frying pan users, 25.8% utilize fresh oil whereas 63.1% reuse oil 2–4 times. A minority of individuals stated reusing oil 4–8 times (8.8%) or more than 8 times (2.3%) (Figure 7). The amount of oil used in the frying pan tended to be small, with alterations appearing quickly. Oil should therefore be changed more frequently. In contrast, electric fryers use a greater volume of oil, leading to more diluted alteration products and, therefore, allowing more foodstuffs to be fried [37]. Only 130 respondents of the present survey used this appliance; four of these indicated that they always use fresh oil, with a higher proportion reusing the oil: 4–8 times (41.5%), more than 8 times (37.7%) and 2–4 times (17.7%).

Factors considered by participants to determine changes to the frying oil are shown in Figure 7B. As shown, the main factors are organoleptic in nature, such as the darkening of the oil (32.0%) and presence of sediments (20.1%). Taste (3.8%) and viscosity (4.2%) were reported to a lower extent. In contrast, 27.8% consider the number of frying cycles used to be the main criterion when determining an oil change. In this sense, Romero et al. [15] reported that Spanish University students usually changed the oil in the electric fryer after a number of frying processes: <5 times (9%), 5–10 times (21%), 11–20 times (25%) and >20 times (8%). Similarly, Gatti et al. [16] indicated that Argentinian adults tended to reuse oil used for domestic practices once (28%), twice (40%), three times (4%) or until some extent of alteration was observed (28%), although they did not specify the type of appliance used. Spanish volunteers mainly clean the oil by filtering it with a strainer (60.1%) and storing it in a closed container (52.6%), open container (14.0%) or in the same frying container (13.2%) (Table 3). Similar habits have been described for adults from Argentina. In this case, the main technique was to use paper to remove excess oil from the food (84%); filter the oil before storage (52%) and store it in the same container (60%), in a glass container (20%) or in a plastic container (20%) [16]. Oil cleaning is also mentioned in acrylamide mitigation strategies during potato frying since removing fines and crumbs is recommended for maintaining frying oil quality [5]. Since frequent use of the same oil for frying potatoes was reported as a household habit of respondents, more emphasis on the importance of oil renewal and its cleaning should be made in communications within the Spanish population.

## 4. Conclusions

In the present study, a survey of domestic practices for cooking and preparing french fries was conducted within the Spanish population. The aim was not to give advice on acrylamide or frying practices but to provide information on domestic habits in relation to french fry preparation, identify which consumer practices may influence acrylamide formation and evaluate compliance with the acrylamide mitigation strategies described by the European Regulation. Results indicated that the Spanish population mainly prepares french fries from fresh tubers acquired in supermarkets (48.1%), preferably in-season potatoes (49.1%), purchased in bulk (36.1%), washed (43.3%) and stored indoors (79.6%). These habits, together with frequent washing of potatoes before frying, are adequate for controlling acrylamide precursor levels (mainly reducing sugar content). However, consumers should be more mindful when selecting the raw material. This is especially the case for those who do not consider the type of potato purchased at market. Improved practices would also include more frequently soaking potatoes to reduce the sugar content of the fresh tubers, particularly during periods when stored potatoes are the only ones available on the market.

Following the typical Mediterranean culinary practices, olive oil was the most commonly used frying oil, followed by sunflower oil, whilst frying pans were the most common utensils used for the frying process, leading individuals to more frequently replace cooking oil. Although most respondents reported controlling frying temperature, advice relating to the maximum temperature recommended for preventing acrylamide formation in french fries should be addressed. On the other hand, examining the color of french fries as the main criteria for deciding the frying end-point was often reported, which coincides with one of the main tips for controlling acrylamide levels and, consequently, exposure to it. In this regard, although a golden color was preferred by respondents, color guidelines should be distributed in order to clarify the definition of “golden.” The population should also be instructed about the introduction of practices in relation to adding salt prior to frying, the type of cut, amount of potatoes in the appliance and cleaning oil after frying in order to understand all possible strategies for acrylamide mitigation at home.

From these results, it might be deduced that culinary domestic habits of the Spanish population are in line with recommendations for mitigating acrylamide during the preparation of french fries. Furthermore, they do not include any practices that increase the risk of acrylamide exposure. Nevertheless, some actions could be mentioned in order to control its presence in this food: (1) educational initiatives including information provision targeting improved domestic behaviors for the promotion of healthy frying habits should be promoted; (2) information should be specially focused on the importance of the selection of suitable fresh tubers; (3) on the washing and soaking of potatoes before frying; and (4) on the control of frying temperatures below 175 °C, avoiding excessive browning of food. All these measures will effectively prevent acrylamide formation and, consequently, will reduce its exposure.

## Figures and Tables

**Table 1:** Sociodemographic characteristics of participants (*n* = 730).

Characteristic	*n*	(%)
Gender		
Male	190	(26.0)
Female	534	(73.2)
Missing	6	(0.8)
Age group		
18–35	209	(28.6)
36–55	395	(54.1)
56–65	86	(11.8)
>65	38	(5.2)
Missing	2	(0.3)
Nationality		
Spanish	714	(97.8)
Other than Spanish	8	(1.1)
Missing	8	(1.1)
Individuals < 18 at home		
Yes	290	(39.7)
No	434	(59.5)
Missing	6	(0.8)
Individuals/home		
1	63	(8.6)
2	217	(29.7)
3	190	(26.0)
4	210	(28.8)
5	33	(4.5)
>5	7	(1.0)
Missing	10	(1.4)
Type of household		
Single	61	(8.4)
Single with children	30	(4.1)
Shared apartment	27	(3.7)
Couple without children	187	(25.6)
Couple with children	313	(42.9)
Couple, children and relatives	16	(2.2)
With parents	26	(3.6)
With parents and siblings	47	(6.4)
Other	12	(1.6)
Missing	11	(1.5)

Number of cases (*n*).

**Table 2:** French fry consumption and characteristics related to the use of fresh potatoes to prepare french fries in a Spanish population (number of cases, (%)).

French Fry Consumption										
	fresh		frozen		not distinguished				missing ^1^	
Type	623	(85.3)	21	(2.9)	84	(11.5)			2	(0.3)
	daily		weekly		monthly		exceptionally		missing ^1^	
Frequency of consumption	2	(0.3)	262	(35.9)	318	(43.5)	144	(19.7)	4	(0.6)
	only french fries		as an accompaniment						missing ^1^	
Consumption	19	(2.6)	709	(97.1)					2	(0.3)
**Fresh Potatoes**										
	neighborhood stores		local markets		supermarkets		hypermarkets		missing ^1^	
Place of purchase ^2^	322	(44.1)	118	(16.2)	351	(48.1)	214	(29.3)	0	(0.0)
	only origin		only variety		both		no		missing ^1^	
Origin/variety	103	(14.1)	84	(11.5)	264	(36.2)	278	(38.1)	1	(0.1)
	yes		no				unknown ^3^		missing ^1^	
Special for frying	113	(15.5)	517	(70.8)			98	(13.4)	2	(0.3)
	bulk		bagged		both		unnoticed ^4^		missing ^1^	
Packaging	268	(36.7)	205	(28.1)	255	(34.9)	0	(0.0)	2	(0.3)
	unwashed		washed		both		unnoticed ^4^		missing ^1^	
Presentation	99	(27.3)	316	(43.3)	171	(23.4)	44	(6.0)	0	(0.0)
	in-season		stored		both		unnoticed ^4^		missing ^1^	
Seasonality	358	(49.1)	31	(4.2)	164	(22.5)	176	(24.1)	1	(0.1)

^1^ Not answered; ^2^ multiple possible answers; ^3^ volunteer does not care about this; ^4^ volunteer cannot be precise.

**Table 3:** Habits for frying potatoes in a Spanish population (number of cases, (%)).

Pre-Frying Stage										
	yes		no						missing ^1^	
Peeled	712	(97.5)	2	(0.3)					16	(2.2)
	yes		no						missing ^1^	
Washed	634	(86.8)	82	(11.3)					14	(1.9)
	yes		no						missing ^1^	
Soaked	185	(25.4)	531	(72.7)					14	(1.9)
	<15 min		15–30 min		>30 min					
If soaking, duration	126	(17.3)	43	(5.9)	16	(2.2)				
	before frying		during frying		after frying		no		missing ^1^	
Salt	263	(36.1)	39	(5.3)	347	(47.5)	79	(10.8)	2	(0.3)
	strips		cubes		chips		irregular		slices	
Type of cut ^2^	642	(87.9)	142	(19.5)	57	(7.8)	85	(11.6)	158	(21.6)
**Frying Stage**										
	frying pan		electric fryer		both		other		missing ^1^	
Kitchen appliance	577	(79.0)	67	(9.2)	63	(8.6)	23	(3.2)	0	(0.0)
	olive		sunflower		both		other		missing ^1^	
Type of oil	574	(78.7)	128	(17.5)	20	(2.7)	7	(1.0)	1	(0.1)
	taste		price		health		performance		appliance	
Criteria for oil selection	375	(51.3)	116	(15.9)	468	(64.1)	132	(18.1)	25	(3.4)
	yes		no		unknown ^3^				missing ^1^	
Special for frying	51	(7.0)	535	(73.3)	144	(19.7)			0	(0.0)
	<half		half		>half		full		missing ^1^	
Potato/appliance surface	59	(8.1)	187	(25.6)	247	(33.8)	235	(32.2)	2	(0.3)
	yes		no		not consumed				missing ^1^	
Defrosts frozen potatoes ^4^	6	(0.8)	100	(13.7)	621	(85.1)			3	(0.4)
	one		two						missing ^1^	
Frying cycles	696	(95.3)	22	(3.0)					12	(1.7)
**Post-Frying Stage**										
	soft		crunchy-soft		crunchy				missing ^1^	
Texture	103	(14.1)	603	(82.6)	24	(3.3)			0	(0.0)
	paper		rack		both		none		missing ^1^	
Removal of oil	424	(58.1)	114	(15.6)	154	(21.1)	36	(4.9)	2	(0.3)
	strainer		paper		decantation		no cleaning		missing ^1^	
Cleaning	439	(60.1)	39	(5.3)	77	(10.6)	164	(22.5)	11	(1.5)
	used appliance		closed container		open container		no reusing		missing ^1^	
Storage	133	(18.2)	384	(52.6)	102	(14.0)	103	(14.1)	8	(1.1)

^1^ not answered; ^2^ multiple possible answers; ^3^ volunteer does not care about this; ^4^ only for frozen par-fried potatoes.

## Supplementary Materials

The following are available online at https://www.mdpi.com/2304-8158/9/6/799/s1, Supplementary material: Survey on household habits in relation to frying potatoes.
