# Supplementary material for: Are Household Potato Frying Habits Suitable for Preventing Acrylamide Exposure?
Source: Foods. 2020 Jun 17;9(6):799. doi: 10.3390/foods9060799 (PMC7353624; doi:10.3390/foods9060799)
Supplement: Supplementary file 1 [file foods-09-00799-s001.pdf]

Survey on Household Habits in Relation to Frying Potatoes

(Translation from Its Original Version in Spanish)

The objective of this questionnaire is to better understand habits in relation to potato frying practices in Spanish households. The survey forms part of the activities carried out as part of the SAFEFRYING project (AGL2015-64234-R) financed by the Ministry of Economy and Competitiveness through the State Plan for Scientific and Technical Research and Innovation. This information will serve to prepare recommendations for the relevant authorities in the field of food safety, companies in the food sector and consumer organizations, with a view to improving food safety for this type of culinary technique.

The information collected in this survey will be treated confidentially. The data obtained will be managed through the CIS Specific Data Bank for Social Studies (ARCES). Data will only be used within the context of the aforementioned research project and access will not be provided to third parties or entities outside of the same.

I. SOCIODEMOGRAPHIC INFORMATION

- City: _____ **Province: _____**

- Gender:

☐ female ☐ male ☐ I prefer not to answer

- Age group:

☐ 18–35 y

☐ 36–55 y

☐ 56–65 y

☐ Above 65 y

☐ I prefer not to answer

- Nationality:

☐ Spanish ☐ Other than Spanish ☐ I prefer not to answer

- Number of individuals at home:

☐ 1 ☐ 2 ☐ 3 ☐ 4 ☐ 5 ☐ more than 5 ☐ I prefer not to answer

- Are there any individuals aged under 18 in your home?

☐ Yes ☐ No ☐ I prefer not to answer

- Type of household. Family situation:

☐ Single

☐ Shared apartment

☐ Couple without children

☐ Couple with children

☐ Single with children

☐ Couple with children and relatives

☐ With parents

☐ With parents and siblings

☐ Other (specify): _____

☐ I prefer not to answer

II. CULINARY HABITS: COOKING EXPERIENCE

1. What experience and level of knowledge do you consider you have in relation to cooking?

☐ None, I never prepare food at home.

☐ I am able to prepare a dish following a simple recipe, although I do not cook regularly (basic).

☐ I am able to prepare some food autonomously, although I do not cook regularly (average).

☐ I have wide experience in cooking and I cook regularly (high).

III. FRENCH FRY CONSUMPTION

2. What type of potato do you use for frying?

☐ Fresh potatoes

☐ Frozen par-fried

☐ Both fresh and frozen par-fried

3. How often do you consume French fries at home? (Do not consider potato chips.)

☐ Every day (daily)

☐ Several times a week (weekly)

☐ Several times a month (monthly)

☐ Several times a year (rarely)

4. How do you usually consume French fries at home? (You may select more than one option.)

☐ As a side dish for meat

☐ As a side dish for fish

☐ As a side dish for vegetables

☐ As a side dish for foods such as eggs, croquettes, sausages, etc.

☐ I like to consume them alone

IV. CHARACTERISTICS OF FRESH POTATOES

5. Where do you normally buy the potatoes you use for frying? (You may select more than one option.)

- ☐ In neighborhood grocery stores
- ☐ In local markets
- ☐ In supermarkets
- ☐ In hypermarkets

6. When you buy potatoes, are you interested in knowing the origin and/or the variety?

- ☐ Yes, but I am only interested in the geographical origin
- ☐ Yes, but I am only interested in the botanical variety
- ☐ Yes, I am interested in both geographical origin and botanical variety
- ☐ No, I am not interested

7. When you buy potatoes for deep frying at home, do you buy potatoes labelled as "special for frying"?

- ☐ Yes
- ☐ No
- ☐ No, I did not know there were potatoes labelled as "special for frying"

8. How are the potatoes you buy normally packaged?

- ☐ Bulk potatoes
- ☐ Bagged potatoes
- ☐ Both bulk and bagged potatoes
- ☐ I do not know

9. With regards to their presentation, what type of potatoes do you usually buy?

- ☐ Unwashed potatoes
- ☐ Washed potatoes
- ☐ Both washed and unwashed potatoes
- ☐ I do not know

10. What type of fresh potatoes do you usually buy?

- ☐ In-season potatoes
- ☐ Stored potatoes
- ☐ Both in-season and stored potatoes
- ☐ I do not know

11. Where do you store fresh potatoes until you are ready to use them?

- ☐ Indoors, inside the home
- ☐ Outdoors, outside the home
- ☐ Both indoors and outdoors

V. PRE-FRYING STAGE

12. Check the practices that you usually engage in prior to the potato frying process. (You may select more than one option.)

- ☐ After peeling the potatoes, I wash them
- ☐ I soak the potatoes in water once peeled before cutting them
- ☐ I soak the potatoes once peeled and cut

13. If you soak the potatoes, how long do you leave them in water?

- ☐ Less than 15 minutes
- ☐ 15–30 minutes
- ☐ More than 30 minutes

14. Do you add salt to the potatoes?

- ☐ Yes, before frying
- ☐ Yes, during frying
- ☐ Yes, after frying
- ☐ No, I do not add salt

15. What kind of cut do you usually perform on potatoes? (You may select more than one option.)

- ☐ Strips
- ☐ Cubes
- ☐ Chips
- ☐ Irregular cut
- ☐ Slices

VI. FRYING STAGE

16. What kitchen appliance do you use to fry potatoes?

- ☐ Frying pan
- ☐ Electric fryer
- ☐ Both frying pan and electric fryer
- ☐ Other type of appliance

17. What type of oil do you usually use for frying potatoes?

- [ ] Olive oil
- [ ] Sunflower oil
- [ ] Other frying oils

18. What is the reason for choosing this frying oil? (You may select more than one option.)

- [ ] Taste
- [ ] Price
- [ ] Health properties
- [ ] Performance of the oil
- [ ] Appliance used for frying

19. Do you use oils identified as "special frying"?

- [ ] Yes
- [ ] No
- [ ] I do not use them and I did not know about the existence of these oils

20. Can you specify the amount of potatoes you fry in relation to the dimensions of the frying appliance (frying pan, fryer basket, etc.)?

- [ ] Less than half of the appliance surface
- [ ] Around half of the appliance surface
- [ ] More than half of the appliance surface
- [ ] To the maximum level of the appliance surface

21. For frozen par-fried potatoes, do you defrost them before frying?

- [ ] Yes
- [ ] No
- [ ] I do not consume frozen par-fried potatoes

22. How do you fry?

- [ ] In one cycle
- [ ] In two cycles

23. How do you usually control the temperature when frying potatoes?

- [ ] I regulate the thermostat (for the electric fryer) or the power of the gas flame, the glass ceramic or the induction plate (for the frying pan)
- [ ] I always select the maximum temperature
- [ ] I do not control the temperature

24. When do you think the oil is ready for frying? (You may select more than one option.)

- ☐ When the oil starts to smoke (smoke point)
- ☐ I do not exercise precise control
- ☐ When the thermostat is turned off
- ☐ I add a potato and watch it start to fry

VII. POST-FRYING STAGE

25. Usually, when do you think the French fries are ready?

- ☐ When the potato acquires the color that I like
- ☐ When the potato stops bubbling in the oil
- ☐ I take out a potato and taste it
- ☐ Other: _____

26. With regards to color, how do you like potato chips?

- ☐ Light-golden
- ☐ Golden
- ☐ Dark-golden

27. With regards to the texture, how do you like potato chips?

- ☐ Soft
- ☐ Crunchy on the outside and soft on the inside
- ☐ Totally crunchy

28. What method do you usually use to remove excess oil from the potato after frying?

- ☐ Drain or shake on a rack or strainer
- ☐ Put the potatoes on absorbent paper
- ☐ Both shake on a rack or strainer and deposit on absorbent paper
- ☐ I do not remove excess oil after frying

29. Approximately how many times do you reuse the same oil to fry potatoes? (Do not select more than one checkbox per row.)

	All the time	2–4 times	4–8 times	> 8 times	I do not reuse
Frying pan	☐	☐	☐	☐	☐
Electric fryer	☐	☐	☐	☐	☐

30. If you reuse the oil after frying the potatoes, how do you usually keep it?

- ☐ In the same frying container
- ☐ In an open container
- ☐ In a closed container
- ☐ I do not reuse frying oil

31. What treatment do you usually use to clean the frying oil?

- ☐ Strainer filtered
- ☐ Paper filtered
- ☐ Decantation
- ☐ I do not reuse frying oil

32. When do you usually think the oil should be changed?

- ☐ When it is dark in color
- ☐ When it has many sediments
- ☐ When it has a strange taste
- ☐ When it has a high viscosity
- ☐ I always change it after a certain number of frying times, regardless of how it looks
- ☐ I always use fresh oil

Thank you for your cooperation.
